# Where Environment and Healthcare Meet: Air Pollution, Antibiotic Use, and Mortality in an Ageing Population in Southern Italy

**DOI:** 10.3390/medsci14020198

**Published:** 2026-04-14

**Authors:** Caterina Elisabetta Rizzo, Roberto Venuto, Maria Gabriella Caruso, Cristina Genovese, Pasqualina Laganà

**Affiliations:** 1 Department of Prevention, Local Health Authority of Messina, 98123 Messina, Italy; roberto.venuto@hotmail.it (R.V.); gabriella.caruso@asp.messina.it (M.G.C.); 2 Department of Chemical, Biological, Pharmaceutical and Environmental Sciences, University of Messina, 98166 Messina, Italy; 3 Department of Biomedical and Dental Sciences and Morphofunctional Imaging, University of Messina, 98124 Messina, Italy; crigenovese@unime.it (C.G.); pasqualina.lagana@unime.it (P.L.)

**Keywords:** One Health, air pollution, antibiotic consumption, mortality, syndemics

## Abstract

Background: Air pollution, antimicrobial use, and population ageing are increasingly recognised as co-occurring pressures shaping population health. This study explores their ecological association with mortality patterns in the province of Messina (Southern Italy), within a One Health-informed framework. Methods: An ecological analysis was conducted using district-by-year data (2015–2024), integrating environmental monitoring (PM_10_, PM_2.5_, NO_2_, O_3_), outpatient antibiotic consumption, and cause-specific mortality rates. Multivariable regression models were used to assess associations between exposures and mortality outcomes. A post-2020 indicator was included to account for COVID-19-related disruption. Results: Marked geographic variability in pollutant concentrations was observed, with higher levels in urban-industrial districts. Infectious disease mortality increased from 13.8 to 44.6 per 100,000 inhabitants between the pre-pandemic and post-pandemic periods. In Poisson regression models, particulate matter showed a small and non-significant association with respiratory mortality (RR = 1.02, 95% CI: 0.89–1.18), while antibiotic consumption was not independently associated with mortality (RR = 0.99, 95% CI: 0.94–1.05). The post-2020 period was associated with higher mortality estimates (RR = 1.15, 95% CI: 0.72–1.83), although with wide confidence intervals. Conclusions: The findings suggest the co-occurrence of environmental, demographic, and pharmaceutical pressures within the same territories, rather than demonstrating formal synergistic interaction. The observed post-pandemic increase in mortality highlights the importance of accounting for COVID-19-related disruption. These results should be interpreted as exploratory, given the ecological design and limited sample size, but support the need for integrated surveillance approaches within a One Health perspective.

## 1. Introduction

Population ageing, environmental degradation, and evolving patterns of antimicrobial use are reshaping the epidemiological landscape of many high-income countries [1]. These dynamics challenge the traditional separation between environmental health, infectious diseases, and chronic disease prevention and highlight the need for integrated analytical frameworks capable of capturing interactions between multiple health determinants [2].

**Figure 1:**
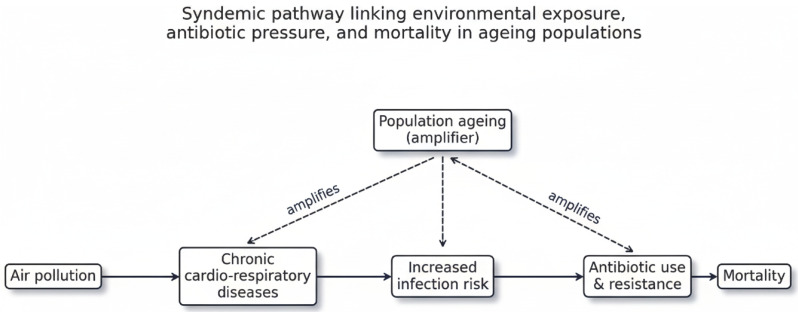
Conceptual framework of the syndemic interactions linking air pollution, infection risk, antibiotic use and mortality in ageing populations, with population ageing acting as a risk amplifier.

Air pollution remains one of the leading environmental risk factors for morbidity and premature mortality worldwide [3]. In Europe, exposure to particulate matter and nitrogen dioxide has been consistently associated with an increased risk of cardiovascular and respiratory diseases through mechanisms involving systemic inflammation, oxidative stress, and endothelial dysfunction [4,5,6]. In addition to its well-documented role in chronic non-communicable diseases, emerging evidence suggests that air pollution may also increase susceptibility to respiratory infections by impairing pulmonary immune defences and facilitating pathogen transmission [7,8,9]. These findings indicate that environmental exposures may influence both chronic and infectious disease outcomes within the same populations. The potential interactions linking environmental exposure, infection risk, antibiotic use and mortality in ageing populations are illustrated in Figure 1.

At the same time, antimicrobial consumption represents another major determinant of contemporary population health [10]. Antibiotics remain essential for the treatment of bacterial infections; however, their widespread use contributes to the development and spread of antimicrobial resistance (AMR), which is increasingly recognised as a major global public health threat [11]. Patterns of antibiotic consumption vary substantially across European countries and regions and are influenced by healthcare practices, prescribing behaviour, and population vulnerability [12]. In populations with higher burdens of chronic disease or environmental exposure, increased infection risk may lead to greater antibiotic use, potentially reinforcing pressures associated with antimicrobial resistance [13].

Population ageing further intensifies these dynamics [14]. Older adults are disproportionately affected by chronic cardiovascular and respiratory diseases, have increased susceptibility to infectious diseases, and are more frequently exposed to healthcare settings where antibiotic prescribing is common [15]. As a result, ageing populations may experience compounded health risks arising from environmental exposures, infectious vulnerability, and healthcare-related factors [16]. These overlapping pressures are particularly relevant in European regions where demographic ageing is advancing rapidly [17].

In recent years, the concept of syndemics has been increasingly used to describe situations in which multiple diseases or health risks interact within specific social and environmental contexts, amplifying their combined impact on population health [18]. Within this perspective, environmental exposures, infectious risks, and healthcare practices can be understood as interconnected components of broader health systems rather than as isolated determinants [19].

Despite growing recognition of these interactions, most empirical studies in Europe have examined air pollution, antimicrobial use, and mortality separately. Few analyses have integrated environmental exposure data, pharmaceutical consumption patterns, and mortality outcomes within a unified analytical framework [20]. This fragmentation limits the ability of public health systems to identify overlapping risk patterns and to design coordinated interventions addressing shared upstream determinants.

The Province of Messina in Southern Italy represents a particularly informative setting for exploring these dynamics. The territory combines several characteristics that are increasingly common across many European regions: rapid population ageing, heterogeneous urban–rural environments, persistent air pollution exposure in metropolitan and industrial areas, and variability in healthcare access and pharmaceutical consumption. These features make it a suitable case study for examining how environmental, demographic, and healthcare-related factors may converge to shape mortality patterns in ageing populations.

Using the Province of Messina as a case study, this study applies a One Health perspective to examine the relationships between air pollution exposure, antibiotic consumption, and mortality over the period 2015–2024. The aim is to identify spatial and temporal patterns linking environmental exposures, pharmaceutical practices, and cause-specific mortality within an ageing population.

Specifically, the objectives of this study were to:

1. Examine the association between ambient air pollution levels and mortality from major chronic diseases.
2. Assess the relationship between antibiotic consumption and mortality from infectious diseases.
3. Explore spatial and temporal overlaps between environmental exposure, antibiotic use, and mortality outcomes within the study area.

By integrating environmental, demographic, and pharmaceutical data, this study seeks to provide empirical evidence relevant to the understanding of emerging health risks in ageing European populations and to inform integrated public health strategies within a One Health framework.

## 2. Materials and Methods

### 2.1. Study Design and Setting

A retrospective population-based observational study was conducted to explore temporal and spatial patterns linking air pollution exposure, antibiotic consumption, and cause-specific mortality within a One Health analytical framework. The study covered the period from 2015 to 2024 and focused on the Province of Messina, located in the Sicilian Region of Southern Italy. The Province of Messina represents a heterogeneous territorial context that includes densely populated urban municipalities, industrial areas, rural inland districts, and insular communities in the Aeolian Islands. This diversity allows the analysis of environmental exposure, healthcare utilisation, and mortality patterns across different territorial and demographic settings within a single administrative unit.

The study design was ecological in nature and aimed to identify population-level associations between environmental, pharmaceutical, and demographic factors rather than to establish individual-level causal relationships.

### 2.2. Mortality Data

Mortality data were obtained from the Italian National Register of Causes of Death (ReNCaM), coordinated by the Istituto Superiore di Sanità and based on official death certificates collected through the national mortality surveillance system.

All deaths occurring among residents of the Province of Messina during the study period were included in the analysis. Underlying causes of death were classified according to the International Classification of Diseases, Ninth Revision, Clinical Modification (ICD-9-CM).

For analytical purposes, deaths were grouped into the following main cause-of-death categories:

- Infectious and parasitic diseases (ICD-9-CM codes 001–139);
- Malignant neoplasms (ICD-9-CM codes 140–239);
- Cardiovascular diseases (ICD-9-CM codes 390–459);
- Respiratory diseases (ICD-9-CM codes 460–519);
- Genitourinary diseases (ICD-9-CM codes 580–629).

Information on age, sex, year of death, and district of residence was used to calculate age-standardised mortality rates (ASMR) per 100,000 inhabitants using the direct standardisation method with the European standard population as reference. Mortality analyses were conducted both at the provincial level and across administrative health districts to capture intra-territorial variability.

### 2.3. Air Pollution Data

Air quality data were obtained from the national air monitoring network coordinated by the Italian Institute for Environmental Protection and Research (ISPRA) and operated locally by the Regional Environmental Protection Agency of Sicily (ARPA Sicilia).

Data were collected from fixed monitoring stations distributed across the province and classified according to European Union air quality monitoring criteria, including urban traffic, urban background, suburban, industrial, and rural stations.

The following air pollutants were included in the analysis:

- Particulate matter with aerodynamic diameter ≤10 μm (PM_10_);
- Particulate matter with aerodynamic diameter ≤2.5 μm (PM_2.5_);
- Nitrogen dioxide (NO_2_);
- Ozone (O_3_).

Annual mean concentrations were calculated for each pollutant to characterise chronic exposure levels during the study period. All measurements complied with quality assurance and quality control procedures required under European air quality directives.

For districts without direct monitoring stations, pollutant concentrations were estimated using spatial interpolation methods based on data from nearby monitoring sites, allowing the derivation of district-level exposure estimates.

### 2.4. Antibiotic Consumption Data

Data on antibiotic consumption were obtained from national pharmaceutical surveillance systems coordinated by the Italian Medicines Agency (AIFA), including reports from the National Observatory on the Use of Medicines (OsMed). Where available, provincial prescription databases were also consulted to refine territorial estimates.

The analysis focused on systemic antibacterial agents classified under the Anatomical Therapeutic Chemical (ATC) classification system code J01.

Antibiotic consumption was expressed using the standardised indicator of defined daily doses per 1000 inhabitants per day (DDD/1000 inhabitants/day), which enables comparison across time and geographic areas.

Consumption patterns were analysed according to:

- district of residence;
- sex;
- age group;
- pharmacological class (e.g., penicillins, cephalosporins, macrolides, fluoroquinolones).

Population denominators used to calculate consumption indicators were obtained from annual demographic estimates provided by the Italian National Institute of Statistics (ISTAT).

### 2.5. Demographic Data

Demographic data were retrieved from ISTAT annual population statistics and included total population size, age distribution, and sex structure for each district within the Province of Messina.

Particular attention was given to population ageing, defined as the proportion of residents aged 65 years and older. This indicator was included in the analysis as a potential modifying factor in the relationship between environmental exposures, antibiotic consumption, and mortality outcomes.

### 2.6. Statistical Analysis

Descriptive analyses were conducted to examine temporal trends and geographic variation across districts. Correlation analyses were performed using Pearson coefficients for approximately normally distributed variables and Spearman rank correlations for skewed variables or small samples. Multivariable regression models were fitted to examine associations between environmental exposures, antibiotic consumption, demographic ageing (percentage of population aged ≥65 years), and mortality outcomes. Given the ecological design and limited sample size, regression analyses were considered exploratory. To account for the disruption associated with the COVID-19 pandemic, a post-2020 indicator variable was included in all models.

To better account for the distribution of mortality data, we fitted Poisson regression models using district-level mortality counts as the outcome and population size as an offset. Given the limited number of district-year observations and evidence of collinearity among predictors, a single-pollutant modelling strategy was adopted. Each exposure variable (air pollution, antibiotic consumption, and demographic ageing) was evaluated in separate models, adjusting for the post-2020 period as an indicator of pandemic-related disruption. Rate ratios (RR) with 95% confidence intervals (CI) were estimated. All models should be interpreted as exploratory.

Collinearity among predictors was assessed using variance inflation factors (VIF), and a single-pollutant modelling strategy was adopted as the primary analytical approach. Model diagnostics included inspection of residuals and identification of influential observations. Given the exploratory nature of the study and multiple comparisons, results were interpreted with emphasis on effect size, direction, and consistency rather than statistical significance alone. All analyses were performed using R version 4.5.3.

### 2.7. Ethical Considerations

This study was based exclusively on aggregated and anonymised secondary data obtained from official administrative and surveillance sources. No individual-level identifiable information was accessed during the analysis.

According to Italian national regulations governing the use of anonymised public health data for research purposes, formal ethical approval was not required.

### 2.8. Methodological Positioning

The analytical approach adopted in this study was informed by the One Health perspective, which emphasises the interconnectedness of environmental, human, and healthcare-related determinants of health. By integrating environmental exposure data, pharmaceutical consumption indicators, and mortality outcomes within a single analytical framework, the study aimed to identify overlapping risk patterns relevant to ageing populations.

Given the ecological nature of the analysis, the findings should be interpreted as population-level associations rather than evidence of causal relationships.

## 3. Results

### 3.1. Air Pollution Patterns Across Districts

**Figure 2:**
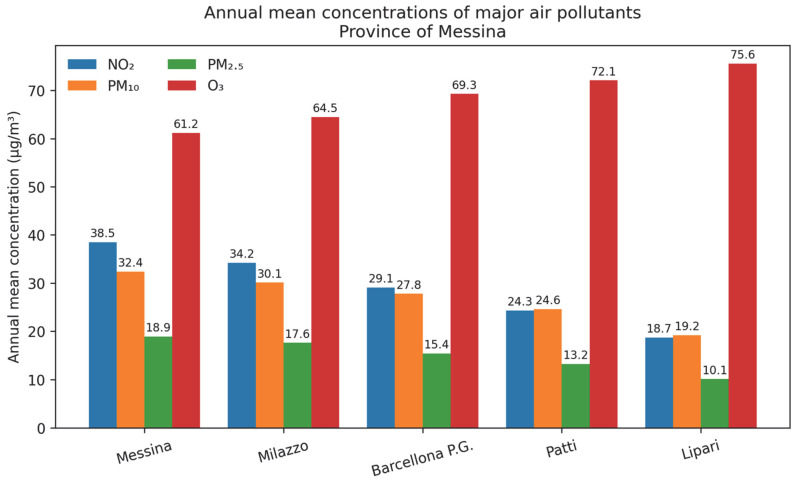
Spatial distribution of major air pollutants across districts of the Province of Messina.

Substantial spatial variability in air pollution levels, as shown in Figure 2, was observed across the Province of Messina during the study period (2015–2024). Urban and industrial districts consistently exhibited higher concentrations of traffic-related pollutants, particularly nitrogen dioxide (NO_2_) and particulate matter (PM_10_ and PM_2.5_), compared with rural mainland districts and insular areas.

Substantial spatial variability in air pollution levels was observed across districts of the Province of Messina during the study period (2015–2024). Higher concentrations of traffic-related pollutants, particularly nitrogen dioxide (NO_2_) and particulate matter (PM_10_ and PM_2.5_), were consistently observed in urban and industrial districts, including Messina and Milazzo, compared with rural and insular areas.

Ozone (O_3_) displayed an inverse spatial pattern, with higher concentrations in rural and insular districts, consistent with its secondary photochemical formation and reduced nitrogen oxide scavenging in less trafficked environments.

Temporal trends showed a non-linear pattern, with a marked reduction in NO_2_ and particulate matter levels in 2020, followed by a progressive increase in subsequent years, approaching pre-pandemic levels.

In exploratory analyses, PM_10_ showed a weak, non-significant correlation with all-cause mortality (r = 0.24; *p* = 0.57), indicating limited statistical power.

### 3.2. Mortality Patterns and Temporal Trends

Between 2015 and 2024, the Province of Messina experienced notable changes in cause-specific mortality patterns. Cardiovascular diseases and malignant neoplasms remained the leading causes of death throughout the study period; however, both categories showed a progressive decline in age-standardised mortality rates. Age-standardised mortality rates for all causes combined showed persistent spatial heterogeneity across districts within the Province of Messina. Cause-specific mortality trends are summarised in Table 1.

Mortality from cardiovascular diseases decreased from 224.0 per 100,000 inhabitants in 2015–2017 to 196.8 per 100,000 in 2022–2024 (−12.1%). Similarly, mortality from malignant neoplasms declined from 193.0 to 162.6 per 100,000 (−15.8%). In contrast, respiratory mortality increased from 42.4 to 62.7 per 100,000 (+47.9%), while infectious disease mortality rose markedly from 12.4 to 44.6 per 100,000 (+259.7%). This increase was concentrated in the post-2020 period.

At the district level, age-standardised mortality rates remained heterogeneous. The metropolitan district of Messina showed higher mortality than the provincial average, reaching 86.23 per 100,000 in 2022–2024. In contrast, lower mortality rates were consistently observed in Lipari (70.32 per 100,000) and Taormina (73.22 per 100,000).

The spatial differences in mortality across districts, shown in Table 2, remained relatively stable over time, measuring 79.99 deaths per 100,000 inhabitants in the period 2015–2017, decreasing slightly to 77.05 per 100,000 in 2018–2020, and increasing to 80.37 per 100,000 in 2022–2024. However, district-level analysis revealed important territorial differences.

The metropolitan district of Messina consistently recorded mortality rates higher than the provincial average, with a notable increase in the most recent period, reaching 86.23 per 100,000 inhabitants. Higher mortality rates were also observed in some inland districts, such as Sant’Agata di Militello and Mistretta, particularly during the earlier periods of analysis, although these areas showed partial convergence toward the provincial average over time.

Conversely, insular and tourist-oriented districts such as Lipari and Taormina consistently exhibited lower mortality rates compared with the provincial average. In Lipari, the age-standardised mortality rate declined from 78.46 per 100,000 inhabitants in 2015–2017 to 70.32 per 100,000 in 2022–2024. A similar downward trend was observed in Taormina. Intermediate mortality levels were observed in the districts of Barcellona Pozzo di Gotto, Milazzo, and Patti, which showed relatively stable trends across the study period.

At the provincial level, the overall age-standardised mortality rate remained relatively stable, measuring 79.99 per 100,000 in 2015–2017, decreasing to 77.05 per 100,000 in 2018–2020, and increasing slightly to 80.37 per 100,000 in 2022–2024.

The metropolitan district of Messina consistently exhibited higher mortality rates than the provincial average, with a notable increase in the most recent period (86.23 per 100,000 in 2022–2024). Elevated rates were also observed in S. Agata di Militello and Mistretta during earlier periods, although these districts showed partial convergence toward the provincial mean over time.

In contrast, Lipari and Taormina consistently recorded lower age-standardised mortality rates, declining from 78.46 and 80.14 per 100,000, respectively, in 2015–2017 to 70.32 and 73.22 per 100,000 in 2022–2024. Barcellona Pozzo di Gotto, Milazzo, and Patti showed intermediate and relatively stable mortality levels across the study period.

### 3.3. Antibiotic Consumption Patterns

Antibiotic consumption showed substantial spatial and temporal variability across the province (Table 3). The reduction in 2020–2021 and subsequent increase likely reflect pandemic-related changes in healthcare utilisation.

Urban districts accounted for a large proportion of overall antibiotic prescriptions, with the metropolitan district of Messina representing nearly half of total consumption within the province. Lower levels of antibiotic use were generally observed in rural and insular districts.

**Figure 3:**
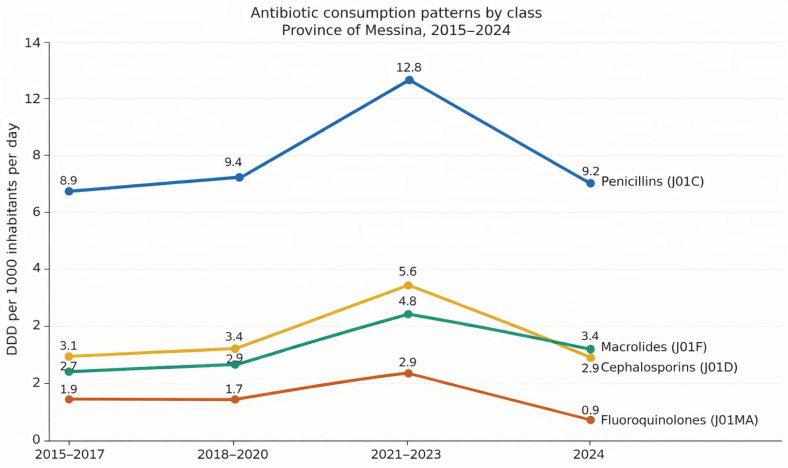
Trends in antibiotic consumption by pharmacological class in the Province of Messina during the study period (2015–2024). Consumption is expressed as defined daily doses (DDD) per 1000 inhabitants per day according to the ATC classification system (J01).

Figure 3 shows that across all districts, penicillins represented the most frequently used class.

Urban areas accounted for a substantial proportion of prescriptions, with the metropolitan district of Messina contributing nearly half of total consumption.

Temporal analysis revealed a reduction in antibiotic use during 2020–2021, followed by a marked increase between 2022 and 2023. Mean consumption increased from approximately 208 DDD per 1000 inhabitants per day in 2021 to 347 DDD in 2023 (+66.8%), before declining slightly in 2024.

Penicillins were the most frequently prescribed class, followed by cephalosporins and macrolides. Higher consumption was observed among older age groups and females.

### 3.4. Integrated Analysis of Environmental Exposure, Antibiotic Use, and Mortality

Spatial comparison of environmental exposure, antibiotic consumption, and mortality revealed the co-occurrence of multiple risk factors within the same districts.

Urban and industrial districts characterised by higher levels of particulate matter and nitrogen dioxide also showed higher antibiotic consumption and higher mortality rates, particularly for respiratory and infectious diseases.

In multivariable regression models, particulate matter (PM_10_) showed a positive but imprecise association with respiratory mortality (β = 0.19, 95% CI: −1.92 to 2.31, *p* = 0.80). Antibiotic consumption was not independently associated with mortality after adjustment (β = −0.01, 95% CI: −0.38 to 0.36, *p* = 0.94).

The post-2020 period was associated with higher mortality estimates (β = 240.49, 95% CI: −718.49 to 1199.46), although with wide confidence intervals.

Collinearity diagnostics showed elevated VIF values for antibiotic consumption (VIF = 26.7) and population ageing (VIF = 28.8), indicating strong interdependence between these variables. These findings support the interpretation of these factors as co-occurring rather than independent synergistic effects. Poisson regression models were fitted using district-level mortality counts with population size as an offset. Each predictor was evaluated in separate models to reduce overfitting (Table 4).

In Poisson regression models, particulate matter (PM_10_) showed a small and non-significant association with respiratory mortality (RR = 1.02, 95% CI: 0.89–1.18, *p* = 0.78). Antibiotic consumption and population ageing were not independently associated with mortality. The post-2020 period was associated with higher mortality estimates (RR = 1.15, 95% CI: 0.72–1.83), although with wide confidence intervals.

Overall, these findings suggest the co-occurrence of environmental, demographic, and healthcare-related factors within the same districts, rather than independent or synergistic effects.

Given the limited number of observations, all statistical analyses should be interpreted as exploratory.

## 4. Discussions

This study provides an integrated ecological analysis of mortality patterns, environmental exposure, and antibiotic consumption in the Province of Messina over the period 2015–2024. By combining environmental, pharmaceutical, and demographic indicators within a single analytical framework, the findings highlight the co-occurrence of multiple pressures shaping population health in an ageing context.

A first key finding concerns the divergence in cause-specific mortality trends. While mortality from cardiovascular diseases and malignant neoplasms showed a progressive decline, respiratory and infectious disease mortality increased substantially in the most recent period [21]. In particular, infectious mortality rose markedly after 2020, a pattern consistent with the documented impact of the COVID-19 pandemic and related excess mortality observed in Italy and other European countries [22]. These results suggest that, despite improvements in the management of major non-communicable diseases, ageing populations remain increasingly vulnerable to respiratory and infectious conditions [23].

The spatial distribution of mortality further supports the presence of territorial inequalities. Urban and industrial districts, particularly Messina and Milazzo, consistently exhibited higher mortality levels compared with rural and insular areas. These differences likely reflect a combination of environmental exposure, population density, and healthcare utilisation patterns rather than a single determinant [24]. Similar urban–rural gradients have been reported in other European settings, where higher exposure to traffic-related air pollution and greater population concentration are associated with increased health risks [25].

Environmental data in this study are consistent with this interpretation. Higher concentrations of nitrogen dioxide and particulate matter were observed in urban and industrial districts, while ozone showed an inverse spatial pattern. Although Poisson regression models did not identify statistically significant associations between particulate matter and mortality outcomes, the direction of the estimates was consistent with existing epidemiological evidence linking air pollution to adverse respiratory and cardiovascular health effects [4,5,6]. The lack of statistical significance is likely attributable to limited statistical power and the ecological structure of the data rather than the absence of a true relationship. In addition, air pollution has been linked to increased susceptibility to respiratory infections through impairment of pulmonary immune responses [7,8,9].

Antibiotic consumption represents an additional dimension of the observed patterns. The temporal trend, characterised by a decline during 2020–2021 followed by a marked increase in 2022–2023, is consistent with pandemic-related changes in healthcare access, diagnostic practices, and prescribing behaviour [26]. Higher levels of antibiotic use in urban districts and among older age groups reflect underlying differences in healthcare demand and vulnerability. However, in multivariable Poisson models, antibiotic consumption was not independently associated with mortality outcomes after accounting for other factors. The widespread use of antibiotics remains a key driver of antimicrobial resistance, a major global public health concern [27].

An important methodological finding of this study concerns the strong collinearity observed between antibiotic consumption and population ageing, as reflected by elevated variance inflation factors. This suggests that pharmaceutical burden and demographic structure are closely intertwined within districts. On this basis, the analysis was conducted using a single-pollutant modelling strategy, and the results are interpreted in terms of co-occurring or converging risk factors rather than independent or synergistic effects.

Taken together, the findings support the interpretation that environmental exposure, healthcare-related factors, and demographic ageing tend to cluster within the same territories. Rather than demonstrating formal syndemic interaction, the results highlight the coexistence of multiple determinants that may jointly shape health outcomes in ageing populations. These findings are consistent with conceptual frameworks emphasising the interaction between environmental exposures, infectious risks, and demographic vulnerability [28,29].

From a public health perspective, these findings reinforce the importance of integrated surveillance approaches [30]. Environmental monitoring, pharmaceutical utilisation data, and mortality outcomes are often analysed separately, yet their convergence within the same populations suggests the need for coordinated strategies [31]. Policies addressing air quality, antimicrobial stewardship, and the health needs of ageing populations may benefit from greater integration within a One Health framework.

Finally, the study highlights the importance of accounting for major external shocks such as the COVID-19 pandemic in the interpretation of temporal trends. The observed increase in infectious mortality and the disruption in antibiotic consumption patterns underline how rapidly population health dynamics can change in response to global events.

Overall, while the results should be interpreted as exploratory, they provide a useful empirical basis for understanding how multiple health determinants may coexist within ageing European populations and for guiding future research adopting more robust longitudinal and spatial analytical approaches.

## 5. Limitations

This study has several limitations that should be considered when interpreting the findings.

First, the ecological design does not allow causal inference at the individual level. Associations observed at the district level may not reflect relationships at the individual level, and the possibility of ecological fallacy cannot be excluded. The results should therefore be interpreted as hypothesis-generating rather than causal.

Second, the number of district-by-year observations was limited, reducing statistical power and leading to wide confidence intervals in regression models. This limitation also constrained the complexity of multivariable analyses and required the adoption of a single-pollutant modelling strategy to reduce overfitting and instability of estimates.

Third, substantial collinearity was observed among key predictors, particularly between antibiotic consumption and population ageing, as indicated by elevated variance inflation factors. This interdependence limited the ability to disentangle independent effects and supports the interpretation of findings in terms of co-occurring rather than synergistic risk factors.

Fourth, exposure assessment was based on aggregated environmental monitoring data, which may not fully capture individual-level exposure variability. Differences in spatial coverage of monitoring stations across districts may have introduced measurement error or misclassification.

Fifth, antibiotic consumption data were derived from outpatient prescriptions and do not capture in-hospital use or the appropriateness of prescribing. Therefore, these data represent an indirect proxy of healthcare-related factors rather than a direct measure of antimicrobial exposure.

Sixth, the study period includes the COVID-19 pandemic, which substantially affected mortality patterns, healthcare utilisation, and antibiotic prescribing. Although a post-2020 indicator was included in the analysis, residual confounding related to pandemic dynamics cannot be excluded.

Seventh, no formal spatial statistical analysis was conducted. While geographic differences were described, the absence of spatial modelling limits the ability to account for spatial autocorrelation and geographic clustering of outcomes.

Finally, although a syndemic framework motivated the study, formal interaction effects between variables could not be robustly assessed due to limited sample size and collinearity. For this reason, the findings are interpreted in terms of co-occurring or converging determinants rather than true synergistic interactions.

## 6. Conclusions

The findings of this study highlight the coexistence of multiple health determinants within the Province of Messina, including environmental exposure, antibiotic consumption, and demographic ageing. While cardiovascular and cancer mortality showed declining trends, increases in respiratory and infectious disease mortality were observed in the most recent years, suggesting an evolving epidemiological profile in ageing populations [32].

The spatial distribution of mortality, air pollution exposure, and antibiotic consumption revealed territorial patterns in which urban and industrial districts experienced higher levels of environmental pollutants and greater pharmaceutical pressure [33]. These findings indicate the co-occurrence of environmental and healthcare-related factors within the same populations, particularly among older age groups more vulnerable to both chronic and infectious diseases [34].

The observed associations between particulate matter exposure and mortality were consistent in direction with existing evidence [35], although not statistically significant in regression models, likely due to limited statistical power. Similarly, changes in antibiotic consumption observed in the post-pandemic period reflect broader European trends and highlight the relevance of antimicrobial use as a contextual determinant of population health [36].

Taken together, these findings support the relevance of integrated analytical approaches capable of examining environmental, pharmaceutical, and demographic determinants within the same population context. Rather than demonstrating formal synergistic or syndemic interactions, the results highlight the convergence of multiple determinants that may collectively influence health outcomes [37].

From a public health perspective, the results suggest that strengthening integrated surveillance systems linking environmental monitoring, antimicrobial consumption data, and health outcomes could improve the identification of emerging risk patterns. Coordinated strategies addressing air quality, antimicrobial stewardship, and the health needs of ageing populations may contribute to improving population health resilience.

Although based on a regional case study, the conditions observed in the Province of Messina—population ageing, heterogeneous environmental exposures, and variable antibiotic consumption—are increasingly common across many European regions. Overall, while the findings should be interpreted as exploratory, they provide a useful basis for understanding the coexistence of multiple health determinants and for guiding future research adopting more robust analytical approaches.

## Figures and Tables

**Table 1:** Age-standardised mortality rates (per 100,000 inhabitants) by major cause of death in the Province of Messina across three time periods (2015–2017, 2018–2020, and 2022–2024). Percentage and absolute changes refer to differences between 2015 and 2017 and between 2022 and 2024.

Cause of Death	2015–2017	2018–2020	2022–2024	Absolute Change	% Change
Cardiovascular	224.0	203.0	196.8	−27.2	−12.1%
Neoplasms	193.0	178.6	162.6	−30.4	−15.8%
Respiratory	42.4	48.7	62.7	+20.3	+47.9%
Infectious	12.4	13.8	44.6	+32.2	+259.7%
Genitourinary	17.1	16.3	13.9	−3.2	−18.7%

**Table 2:** Age-standardised mortality rates (per 100,000 inhabitants) by district in the Province of Messina for the periods 2015–2017, 2018–2020, and 2022–2024.

District	2015–2017	2018–2020	2022–2024	Absolute Change	% Change
Province overall	79.99	77.05	80.37	+0.38	+0.5%
Messina	79.37	77.75	86.23	+6.86	+8.6%
Barcellona P.G.	78.72	79.27	77.32	−1.40	−1.8%
Milazzo	78.81	76.40	75.65	−3.16	−4.0%
Mistretta	83.51	71.81	78.46	−5.05	−6.0%
Patti	77.97	75.68	77.23	−0.74	−0.9%
Taormina	80.14	74.85	73.22	−6.92	−8.6%
Lipari	78.46	71.77	70.32	−8.14	−10.4%
S. Agata	84.99	77.59	76.81	−8.18	−9.6%

**Table 3:** Temporal trends in antibiotic consumption (DDD per 1000 inhabitants/day).

Year	Mean Consumption	% Change vs. Previous Year
2020	259.05	—
2021	208.23	−19.6%
2022	263.12	+26.4%
2023	347.71	+32.2%
2024	340.87	−2.0%

**Table 4:** Exploratory Poisson regression analysis of respiratory mortality (district-by-year observations).

Predictor	Rate Ratio (RR)	95% CI (Lower)	95% CI (Upper)	*p*-Value
PM_10_ (per 10 µg/m^3^)	1.02	0.89	1.18	0.78
Antibiotic consumption (per 100 DDD)	0.99	0.94	1.05	0.91
Population aged ≥65 (%)	0.98	0.90	1.07	0.62
Post-2020 period (COVID indicator)	1.15	0.72	1.83	0.54

## Data Availability

The original contributions presented in this study are included in the article. Further inquiries can be directed to the corresponding author.
